# Experiences and Outcomes of Peer Navigation and Support Interventions for Adolescents on HIV Treatment in Sub-Saharan Africa: A Qualitative Evidence Synthesis

**DOI:** 10.3390/ijerph23040488

**Published:** 2026-04-13

**Authors:** Bernard Nhlanhla Mabuza, Charné Petinger, Brian van Wyk

**Affiliations:** School of Public Health, University of the Western Cape, Cape Town 7535, South Africa; 3720520@myuwc.ac.za (C.P.); bvanwyk@uwc.ac.za (B.v.W.)

**Keywords:** HIV, peer navigation and support, adolescents, retention in care, qualitative evidence synthesis, Sub-Saharan Africa

## Abstract

**Highlights:**

**Public health relevance—How does this work relate to a public health issue?**

- Sub-Saharan African (SSA) adolescents living with HIV (ALHIV) still encounter low viral load suppression (VLS) rates, low treatment adherence and substandard engagement in care, all of which showcase the major barriers encountered by ALHIV.
- The review estimated that about 90% of the predicted 1.75 million ALLHIV globally reside in SSA, where intersecting structural, psychosocial, and biological vulnerabilities tend to increase their risk of treatment interruption, loss to follow-up, and poor mental health outcomes.

**Public health significance—Why is this work of significance to public health?**

- This review showcases the significance of employing peer navigation interventions across SSA in order to improve the quality of lives of ALHIV and the delivery of HIV care.
- The review showcases the roles played by peer navigators (PNs) for ALHIV, and how this serves as a foundational mechanism for ART normalization, trust and credibility. The review also highlights the consistent gaps in the deployment of PNs, which in turn undermine the effectiveness of PN interventions.

**Public health implications—What are the key implications or messages for practitioners, policy makers and/or researchers in public health?**

- Practitioners have to adopt hybrid and flexible delivery modes to deliver services, integrating in-person and online engagement, such as WhatsApp/SMS to accommodate ALHIV’s conflicting demands with schooling, issues around mobility and access, and dynamic preferences about communication in the interest of maintaining continuity of care.
- Researchers and policymakers need to provide formal guidance (and guidelines), supervision structures that are supportive, and adequate remuneration for PNs. Program implementers should also explicitly measure and define the mechanisms of change (such as trust-building) to prevent burnout of PNs, support sustainability, and strengthen the evidence base for the scaling up of peer navigation programs.

**Abstract:**

Adolescents living with HIV (ALHIV) face ongoing challenges with treatment adherence and engagement in care, resulting in lower viral suppression rates compared to adults. Peer navigation has shown promise in supporting psychosocial well-being and adherence among adults, but evidence specific to adolescents in sub-Saharan Africa (SSA) remains limited. This qualitative evidence synthesis (QES) describes and assesses the quality of qualitative and mixed-methods studies on peer navigation and support interventions for ALHIV receiving antiretroviral therapy in SSA. Eligible studies, published in English between January 2015 and October 2025, were identified through a comprehensive search strategy in PubMed, Scopus, CINAHL, and APA PsycArticles. Data were extracted and analyzed thematically using Atlas.ti, and aligned with the Context–Intervention–Mechanism–Outcome (CIMO) framework. PNs in the studies were young people living with HIV who provided education, counselling and adherence support to their peers who were ALHIV. Effective programmes featured structured training, supportive supervision, and flexible delivery models adapted to adolescents’ preferences. Mechanisms of change included trust-building, emotional support, disclosure coaching and empowerment. Reported outcomes included improved adherence, clinic attendance and various psychosocial indicators. However, challenges such as stigma, role ambiguity, limited remuneration, and lack of policy guidance constrained the sustainability and scalability of PN programs. Overall, peer navigation interventions appear effective in strengthening adolescent HIV care when PNs are adequately trained, supervised and contextually adapted. The variation in how peer navigation and support interventions for ALHIV are delivered and designed, along with the lack of standardization of the interventions, may affect the generalizability of the findings and the rollout of PN programs across SSA.

## 1. Introduction

Global reports from UNAIDS indicate that around 1.75 million adolescents aged 10 to 19 years are affected by HIV, with approximately 90% of this population living in sub-Saharan Africa (SSA) [1]. The World Health Organization (WHO) revised the 95–95–95 global targets—that 95% of those living with HIV are aware of their status; 95% of the people diagnosed with HIV receive treatment; and 95% of those on HIV treatment achieve viral suppression—with the intention of ending the HIV/AIDS epidemic by 2030 [2]. While global reports indicate that viral suppression among adults living with HIV exceeds 90%, adolescents living with HIV (ALHIV) demonstrate a considerably lower viral suppression level of 64% [1,2]. In SSA, it is widely agreed that biological, structural and physical challenges experienced by ALHIV lead to poor psychological well-being, and low levels of viral suppression, engagement in care and ART adherence [3,4,5,6,7]. The need to implement adolescent-specific interventions to improve viral suppression and ART adherence and promote mental well-being are recommended [8]. These include various peer-led approaches (including peer navigation and support interventions) to strengthen psychosocial support, adherence and engagement in HIV care in ALHIV [9,10,11,12,13].

In the current review, *peer navigation* refers to structured support provided by peer navigators (PNs), who are referred to as trained individuals with a lived experience of HIV who provide mentorship and psychosocial support to adolescents within a healthcare system [13]. Murphy and colleagues defined peer support as emotional and social support that is offered and then received by people who share the same experience [14]. Peer navigation and support signifies a patient navigation approach that is designed to help clients (i.e., ALHIV) navigate and access complex healthcare services, while simultaneously offering clients psychosocial guidance with the intent of enhancing mental well-being [14]. Current evidence from various reviews reports peer support and support-based interventions as effective for adult populations [10]. PNs enhance patient support as they serve as positive role models for their clients, guiding them towards mitigating barriers to ongoing treatment and care [15]. PNs further offer accurate and pertinent information with practical strategies aimed at supporting sustained engagement in care and treatment adherence [16]. However, there is a lack of evidence linking peer navigation and support intervention to improved retention in care, ART adherence or mental well-being for ALHIV, particularly in SSA [17,18].

Several studies highlight the positive impacts of peer navigation and support interventions for ALHIV in Nigeria, Zambia, Zimbabwe, Kenya, Tanzania and Eswatini [19,20,21,22]. The above studies demonstrated that PNs are effective in mitigating stigma and in improving resilience and independence amongst ALHIV [18,21,23,24,25]. Nevertheless, peer navigation and support interventions are complex, with a lack of clarity and poor description, leading to inconsistent outcomes, flagging the need of a further detailed in-depth study [26]. There are inconclusive results yielded by systematic reviews focusing on peer support and peer navigation interventions for the wider population of people living with HIV (including ALHIV), highlighting the complexity and variability involved in implementing these interventions [27,28]. This qualitative evidence synthesis aimed to provide a contextual understanding of the implementation of peer navigation and support programs for ALHIV in SSA, and unpack the key mechanisms of the interventions that are necessary for positive outcomes. As already indicated, several reviews have explored peer navigation and support interventions for people living with HIV with a focus on adults [26]. However, adolescents and youth are mostly underrepresented in these reviews, because older adolescents are grouped together with adults, thereby masking the effects of these interventions on adolescents specifically [18]. Furthermore, the abovementioned review considered only the quantitative studies of effects, and thereby provides scant description or explanation of how the intervention was delivered or how the mechanism of delivery contributed to the reported results (or their variability). This review is an extension of the qualitative evidence synthesis (QES) protocol previously published by Van Wyk et al. [29].

This review extends the existing literature, as it first focuses on ALHIV in SSA, since previous studies often sampled youth or adult samples. Secondly, the review not only focuses on the outcomes of the synthesis by explicitly analyzing how peer navigation operates, but draws on the CIMO-informed framework to clarify the underlying mechanisms that link intervention components to outcomes. Thirdly, the review consolidates implementation challenges and enablers across diverse settings in SSA, therefore providing practical insights to inform the adaptation, design and scaling up of peer navigation and support interventions for ALHIV. This review provides a detailed understanding of the mechanisms through which outcomes and measures of effectiveness of peer navigation and support interventions are achieved, with implications for the development of future interventions. Additionally, the qualitative synthesis highlighted common challenges of peer navigation and support interventions as experienced by adolescents and young adults living with HIV(AYAH) across different contexts. Therefore, the evidence generated from this review can guide implementation in diverse settings and support greater effectiveness of peer navigation and support interventions in improving treatment outcomes. This review calls for the implementation of hybrid and flexible models of peer navigation that prioritize confidentiality, trust-building, and communication preferences of adolescents. Thus, policymakers should call for the integration of peer navigation into national adolescent HIV strategies through standardized guidelines, structured supervision and sustainable compensation for PNs. Additionally, researchers should explicitly measure mechanisms of change and comprehensively report implementation processes to support scalability, replication, and the longer-term sustainability of youth-friendly tailored programs.

## 2. Materials and Methods

### 2.1. Registration

This qualitative evidence synthesis was prospectively registered with PROSPERO (registration number CRD42024541951) and the protocol that describes the review’s questions, aims and objectives and methods was published [28]. The review adhered to the PRISMA 2020 guidelines [30] and the ENTREQ reporting standards for qualitative reviews [29].

### 2.2. Review Questions

The review addressed the following two primary questions, as described in the protocol [29]:

- What are the experiences and outcomes of peer navigation and support intervention for adolescents and young adults on HIV treatment in sub-Saharan Africa?
- What are the mechanisms used/triggered in delivering peer navigation and support interventions?

The review aimed to synthesize the literature on the delivery, outcomes, and experiences of peer navigation and support intervention for AYAH in sub-Saharan Africa [29]. The objectives of the review were as follows:

- To explore the uptake and barriers experienced in AYAH in peer navigation and support interventions.
- To review the mechanisms by which peer navigation and support interventions are delivered in order to effect behavior change.
- To synthesize the qualitative evidence on the outcomes of peer navigation and support intervention on engagement in care, adherence, viral load suppression and mental wellness for AYAH in sub-Saharan Africa [29].

### 2.3. Information Sources

This QES drew on searches conducted across multiple electronic databases, including PubMed, Wiley Online Library, EBSCOhost (PsychARTICLES, Scopus, MEDLINE, and CINAHL), and Google Scholar. The grey literature (e.g., theses, reports, and conference abstracts) was excluded from the search strategy, because the review specifically aimed to synthesize evidence on the outcomes of peer navigation and support interventions for ALHIV [29].

### 2.4. Search Strategy

An initial search of the PubMed database was undertaken to identify relevant MeSH terms using controlled vocabulary and potential search terms. Consultation with a health information specialist further supported the refinement and adaptation of the search strategy for use across various databases. The search strings utilized in this review can be seen in Box 1 [29].

Box 1 **Search strings.**

(“HIV” OR “AIDS” OR “antiretroviral therapy”) AND

(“ALHIV” OR “Adolescent” OR “teenagers” OR “young adults” OR “youth”) AND

(“Sub-Saharan Africa”) AND

(“Peer supporter” OR “peer navigator” OR “peer mentor”) AND

(“Peer navigation and support intervention” Or “Effectiveness” Or “Outcomes” Or “Experiences”)

### 2.5. Inclusion Criteria

The review included qualitative and mixed-methods studies published between 2015 and 2026, unfolding the experiences of peer navigation interventions (C—concept) for adolescents living with HIV (ALHIV) (P—participants) conducted in sub-Saharan Africa (C—context) [29]. The review only considered studies that were published in English. As seen in Table 1, the Population, Concept and Context (PCC) framework was used to guide the search strategy [29].

### 2.6. Exclusion Criteria

The exclusion criteria for this review included the following: all of the different types of reviews, studies that were conducted outside SSA, solely quantitative studies, studies in which adolescents were not the primary/main beneficiaries of the intervention, and studies that were non-English. Among the excluded studies are those that were published before 2015 and the grey literature.

### 2.7. Study Selection

**Figure 1:**
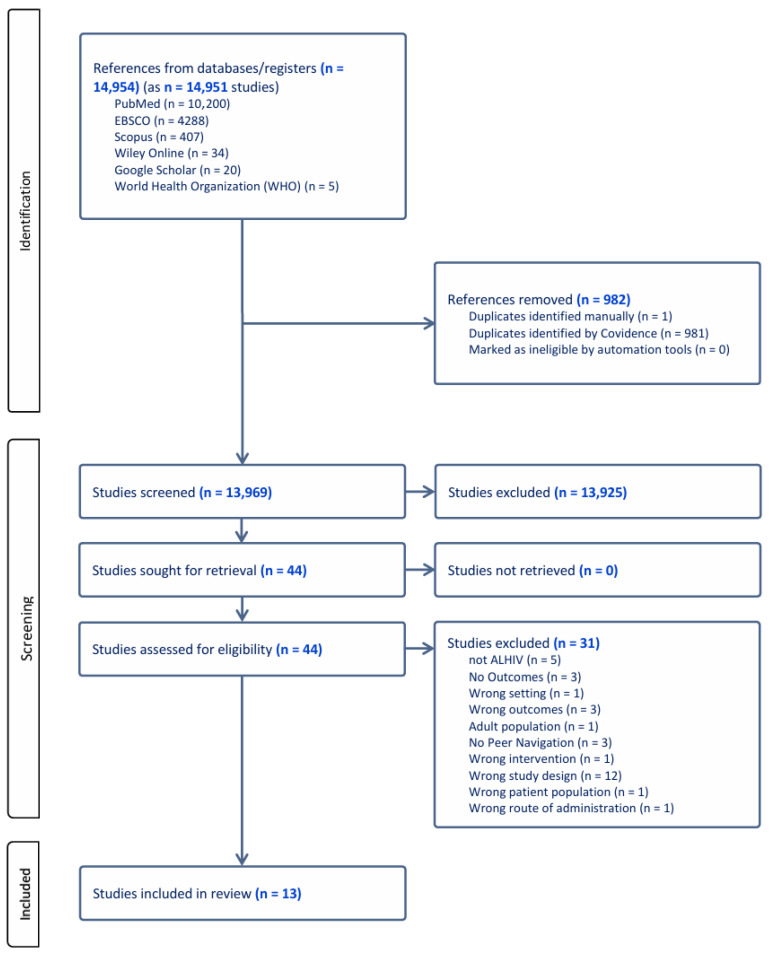
Prisma flow diagram. 3 articles were merged as they speak to the same intervention, as Merrill et al. [31] was merged with Burke et al. [32]. Simms et al. [20] was merged with Bernays et al. [26] and Wogrin et al. [27].

The study selection followed the procedure described in the protocol [29]. Two reviewers independently screened the titles and abstracts of potentially eligible studies. Full-text screening of studies meeting the eligibility criteria was then conducted independently by the same two reviewers to ensure that there is accuracy in study inclusion. Any disagreements regarding inclusion were resolved through discussion between the two reviewers (BNM and CP) until consensus was reached. For quality appraisal, both reviewers assessed all included studies, with further consultation and oversight from the senior reviewer (BvW). The PRISMA flow diagram (Figure 1) was used to report on each stage of the study selection process. For the review, adolescents are defined as individuals aged 10–19 years, while young adults are defined as being between the ages of 20 and 24 years. The review includes young adults because young adults often continue to access adolescent youth-friendly HIV services and encounter similar challenges as adolescents when transitioning to adult care.

### 2.8. Data Extraction

Data extraction was conducted in a step-by-step process using thematic synthesis, as described in the protocol [29]. Firstly, direct quotes that described or explained participants’ experiences of the intervention were extracted from each included study. These direct quotes represented the first-order data. Secondly, following extraction, the quotes were coded and categorized into themes and sub-themes. Data extraction was then guided by the themes and sub-themes reported in the included studies, which are also referred to as code groups and codes in Atlas.Ti (Version 23) software. The themes and sub-themes from the first included study were used as a reference framework for extracting and coding data from the remaining studies.

The initial step involved naming the code groups (themes) based on the findings of the first study. Data from the second and subsequent studies were then compared with these initial themes. Where themes and sub-themes had similar meanings and names, the themes were retained without any changes. When the meaning was similar but the naming differed, the most appropriate theme or sub-theme name was selected and applied consistently across studies. In cases where the extracted data did not fit within the existing themes, new themes or sub-themes were created. When a theme contained different meanings, it was then divided into separate themes and given new names that more accurately reflected the meaning of the data. Overall, these meta-synthesis steps reflect a thematic synthesis across all the included and reviewed studies, with consolidated themes (code groups) and sub-themes (codes) consistently labelled throughout data extraction and analysis.

### 2.9. Quality Assessment

This review employed the Critical Appraisal Skills Program (CASP) tool to assess the methodological quality of the included purely qualitative studies [29]. For studies using mixed-methods designs, the qualitative components were evaluated using the Mixed-Methods Appraisal Tool (MMAT) [33]. Both the MMAT and CASP tools ensured that the appraisal criteria were appropriately aligned with each study design, while still enabling consistent evaluation across diverse studies.

### 2.10. Assessment of Confidence in Review Findings

Grading of Recommendations Assessment, Development and Evaluation Confidence in Evidence from Reviews of Qualitative Research (GRADE CERQual) was used to assess the confidence in the review findings [34]. We assessed four key components: methodological limitations, coherence, adequacy of contributing data and relevance [29]. Initial confidence grading was assigned ratings using GRADE CERQual across four components (coherence, methodological limitations, relevance, and adequacy). Two reviewers independently evaluated and rated the review findings (high, moderate, low, or very low confidence), followed by all reviewers working together to reach a final agreement on the grading decision.

### 2.11. Data Management, Analysis and Synthesis

Covidence software was utilized during all the stages of this review process, including literature searches, study selection, data extraction, quality appraisal, and narrative synthesis, as described in the protocol for the review [29]. Covidence was also employed for data management and the screening of included studies. The data were analyzed using thematic synthesis with Atlas.Ti (Version 25), which informed the identification and description of key themes and overall review findings. Thematic synthesis involves systematically reviewing findings by clustering codes into descriptive themes, which are then refined into analytic themes [34]. Therefore, Atlas.Ti was used to assist with the review’s analytical process, including systematic coding and theme generation. Where feasible, Context–Intervention–Mechanism–Outcome (CIMO) configurations were developed for sufficiently homogeneous subgroups of studies as part of a second-order analysis. The CIMO configuration was defined by Denyer [35] as a framework for explaining how an intervention (I), applied in a certain context (C), triggers specific mechanisms (M) that lead to the outcomes (O) of interest.

## 3. Results

### 3.1. Description of Included Studies

Table 2 summarizes the characteristics of the included studies. In total, 16 articles were identified for extraction. To reduce duplication, articles reporting on the same intervention were merged: the Zvandiri–Friendship Bench cluster [20], with companion papers [26,27] and the Project YES! trial [31,32]. Following this process, the dataset reflects 13 unique studies.

Three studies were conducted in Zimbabwe, South Africa and Kenya, while two were in Eswatini, and one was in Nigeria. Sample sizes ranged from 10 to 842 participants. Most studies (N = 9) enrolled adolescents and young adults (aged 12–24 years) living with HIV (AYAH) [12,16,31,36,38,39,40,41,42], while three papers focused on peer navigators (PNs) as study participants [18,20,37]. In all studies, AYAH was defined as between 10–24 years old and engaged in HIV treatment. In two studies, key informants such as nurses, programme coordinators and/or caregivers were included [20,40].

In terms of study design, nine used qualitative methods [12,31,36,37,38,39,41,42]; and two employed mixed-methods designs [20,36]. Two qualitative studies were embedded in a randomized controlled trials (RCT) [21,40].

Data collection methods included mostly focus group discussions (FGDs) in seven studies [16,20,31,39,40,41,42]; followed by semi-structured or in-depth interviews in six studies [16,31,38,39,41,42]; with case review notes in one study [42]. Quantitative data collection involved close-ended questionnaires, self-reported assessments, and mental health screening tools such as the Shona Symptom Questionnaire (SSQ), the Patient Health Questionnaire-9 (PHQ-9), and the EQ-5D [21,41,42], in addition to routine clinical data on antiretroviral therapy (ART) initiation, retention, and viral load completion or suppression [36] The current synthesis focused on the qualitative studies and components only.

### 3.2. Description of Peer Navigation and Support Interventions

Table 3 summarizes the key characteristics of peer navigation interventions across all studies. Each study referred to PNs differently: with three named PNs [19,38,39], four peer mentors or young peer mentors (YPM) [31,36,37,41], three as Community Adolescent Treatment Supporters (CATS) [12,20,42], two referred to as expert clients (ECs) [16,17], and one study referred to them as lay counsellors [40]. PNs are generally described as youth living with HIV, ranging between 18 and 28 years [12,20,31,38,42]. PNs continuously draw on their lived experiences to provide effective peer support, education, mentoring, counselling and community-based tailored adherence support to adolescents living with HIV [12,17,19,20,42].

#### 3.2.1. Training of PNs

The training of the PNs was varied across studies, with training periods of one day to one month [19,31,36,38,40], while other studies did not specify the training period [12,16,17,37,39,41,42]. In six studies, PNs received training to develop their empathy, active listening, communication, and encouragement skills to provide peer support [12,16,17,19,37,41]. The training focused mostly on equipping PNs with the ability to facilitate, utilize open-ended questions during communication, conduct non-judgmental communication, and foster safe and supportive environments for adolescents [12,39]. PNs were also trained to provide to provide problem solving therapy (PST) steps, which incorporate problem identification, brainstorming solutions, and action planning [20].

Apart from theoretical training, PNs received practical training through practice sessions and role-plays [20], and through using methods such as inquiry-based stress reduction (IBSR) methods, meditation, journaling, creativity and facilitating [42]. The content of the training workshop or curriculum addressed topics such as HIV/AIDS and treatment education/literacy, youth-friendly services, common myths, privacy and confidentiality, sexual health, and ethical issues [12,19,37,38,39,40]. PNs were also trained in maintaining and neutralizing conflict between participants, suicidal thoughts, and the spreading of myths by adolescents. Different measures were used to deliver peer support across the interventions.

#### 3.2.2. Types of Interventions

Across all studies, seven interventions were delivered through a combination of in-person and electronic engagement with participants [12,19,20,36,38,39,42]. Five were conducted in-person only [16,17,31,37,40], and one was delivered online/electronically via text-messaging, WhatsApp, and call [41].

Peer navigation was delivered through social platforms, text messages, calls, and automated messages, where the content of the messages focused mainly on encouragement, clinic scheduled visit reminder, and offering counselling. For example, in the E-NAV intervention, PNs utilized a combination of calls, WhatsApp messages, and automated messages to provide adolescents with HIV education and psychosocial support, in an attempt to improve ART adherence and engagement in clinics [12,19,20,38,39,41]. PNs conducted peer-to-peer support groups, virtual support groups, and in-person individual engagement in clinic visits in order to allow adolescents to freely discuss their challenges and needs flexibly through texting or in-person [40,41].

Electronic or online interventions were conducted through the utilization of mobile phones to call, SMS text and WhatsApp message, as PNs received training on those devices prior to the initiation of the interventions [12,16,17,20,36,38,39,40,41]. In-person interventions occurred within health facilities on days when adolescents had scheduled appointments to collect their treatment or had to attend teen or youth-adherence clubs [12,16,17,19,20,31,36,37,38,39,40,42].

Beyond helping peers in a clinical setting, PNs went beyond to ensure that adolescents are re-engaged in adolescents lost to follow-up, through the provision of literacy linked to social health literacy [12,16,17]. PNs also promote support on community-based adherence support, and following up on home visits for those who missed appointments by seven to nine days, to improve treatment continuity [17]. During the implementation of peer navigation interventions (e.g., Zvandiri), PNs also document all sessions and identify red flags (e.g., suicide ideation) escalating during the intervention [20]. Additionally, PNs make sure that pills are being taken by counting how many pills have been taken post-last refill appointment [17].

#### 3.2.3. Intervention Outcomes

The central outcome of the interventions was to increase ART initiation, viral load suppression, and adherence, while ensuring that adolescents are given the platform to comfortably discuss issues of HIV and gain knowledge in treatment services [19,36,38]. Most of the interventions sought to ensure that adolescents are retained in care, receive treatment, gain independence, and are resilient towards taking their HIV treatment, and simultaneously improve their treatment outcomes [16,17,39]. The involvement of peer supporters and caregivers in some of the interventions led to an increase in self-acceptance and fostered relationships for adolescents [31,40]. Lastly, the interventions also utilized positive health support to overcome adolescents’ experiences of social isolation, stigma, and confidentiality concerns, to improve adolescents’ self-worth, communication, emotional well-being, and body positivity [36,41,42]. Overall, the intervention’s main focus was to reduce viral load and ensure that adolescents living with HIV are retained in care, and provide psychosocial support in helping them overcome their barriers/struggles. Across the studies, the PNs receive training that is sufficient to allow them to effectively provide peer support to adolescents, and further increase adolescents’ viral load suppression and retention in care, and reduce their psychosocial issues. The function of PNs was homogeneous across all studies. However, even though the interventions carried out one mission of providing youth-centered and peer-driven support to adolescents, the interventions were heterogeneous in terms of their methods and in their mode of delivery. The interventions also differed in terms of structure and settings, and their primary focus, as they varied from focusing on mental health, reduction of stigma, disclosure, and adherence.

### 3.3. Thematic Framework

Before conducting formal coding, we undertook an initial interpretive review of the findings across all included studies to identify recurring concepts and areas of convergence. This preliminary phase involved examining reported results, author interpretations, and intervention descriptions to map emergent thematic domains relevant to peer navigation implementation. Rather than applying an a priori framework, we allowed themes to surface inductively through repeated engagement with the data. These early thematic areas served as the foundation for organizing the synthesis and informed the subsequent alignment with the Context, Intervention, Mechanism and Outcome (CIMO) framework. This preparatory step ensured that the configurations presented in Table 4 were grounded in the evidence and reflective of the diversity of implementation contexts reported across studies.

#### 3.3.1. Foundations of Peer Navigation

This theme focuses on the significant peer navigation components, the examination of PNs focusing on their identity, role, and significance. Throughout the contexts, peer navigation has its foreground on shared lived experience, role modelling, and relatability [12,16,17,19,20,31,36,37,38,39,41,42]. These qualities serve as the foundation for credibility and trust between PNs and adolescents, while also defining the identity and makeup of the peer navigation workforce (Intervention) [39,40].

#### 3.3.2. Training and Preparation

Training of PNs helps equip them with effective competence towards delivering support, counselling skills, confidentiality practices, and HIV literacy [12,16,19,36,37,38,39,40,41,42]. However, the prevalent absence of organized mentorship and standardized curricula may sometimes undermine the PNs’ consistency and clarity of their role [16,26]. This training of PNs serves as a key mechanism that enables the building of skills and confidence before the implementation of the intervention [16,31].

#### 3.3.3. Supervision and Support Systems

This theme reflects on the contextual conditions that are significant to peer navigation program sustainability. Supervision through the provision of emotional support, case reviews, and ongoing mentorship has a significant role in enhancing PNs’ well-being and role performance [16,17,31]. In contrast, emotional strain and insufficient oversight may lead to PNs’ experience of burnout, consequently ruining the quality and continuity of the peer navigation program [20,38,42].

#### 3.3.4. Delivery Modalities and Flexibility

The accessibility and engagement with the peer navigation intervention were directly influenced by the peer interaction frequency and mode of delivery (in-person, mobile, or hybrid) [19,36,39]. Adaptation to the manner in which adolescents prefer to communicate daily ensured participation and consistent responsiveness from the adolescents [19,36,41]. The mode in which the delivery and design of peer navigation intervention affects the activation of the mechanisms of change in different contexts [19,38,39,41].

#### 3.3.5. Mechanisms of Change

This theme focuses on unpacking the reason (why) peer navigation functions. The mechanisms comprise support (emotional and informational), empowerment, trust-building, and shared experience [12,31,39,40,41,42]. PNs’ psychological and social methods enhance self-efficacy and esteem, adherence, and resilience, which illustrates the exclusive value of delivering peer interventions in comparison with routine care (Mechanism) [12,31,39,40,41,42].

#### 3.3.6. Outcomes and Impact

Peer navigation interventions significantly contribute to the psychosocial outcomes of AYAH [19,31,37,38,39,40]. As the intervention improves on clinic attendance, viral suppression, and ART adherence, accompanied by improved resilience, self-worth, and stigma reduction [12,20,31,40,41]. Overall, peer navigation intervention outcomes highlight the effectiveness of creating well-designed systems of peer navigation in translating the mechanisms of support to concrete health benefits.

When put together, these CIMO-associated themes highlight how peer navigation interventions for AYAH function in sophisticated and ever-changing systems. This synthesis offers a comprehensive knowledge of what works, for whom, and under what circumstances through evaluating the interaction between contextual factors, mechanisms of change, intervention design, and consequent outcomes. The insights are significant in refining models that exist, guiding the strategies of implementation, as well as informing policy in strengthening peer navigation programs within high HIV-prevalence and limited resource settings.

### 3.4. Appraisal of Included Studies

#### 3.4.1. CASP Appraisal

The review utilized the CASP tool in assessing the quality appraisal of nine purely qualitative studies [12,16,17,31,37,38,39,40,42]. The results gathered through the CASP checklist are presented in Table 5. The checklist revealed that all studies had provided a clear statement of the aims and objectives of the research, along with an appropriate qualitative methodology. All studies have utilized an appropriate research design to address the aims of the research, as well as an appropriate data collection method in addressing the research questions. For seven studies, the relationship between the participant and the researcher was not clearly stated, while two of the studies clearly stated the relationship between the researcher and participants [38,39]. Moreover, all studies adhered to ethical considerations, conducted sufficiently rigorous analyses, and further stated the findings clearly. Overall, all the studies provided valuable research insight that can be utilized in transforming and informing future interventions on implementing peer navigation and support, which can aid in supporting ALHIV in care.

#### 3.4.2. MMAT Appraisal

This QES utilized the MMAT for the quality appraisal for the mixed-method studies, and the results are summarized in Table 6. The checklist findings revealed that all four studies provided clear research questions, with data being collected adequately to address the research questions. All qualitative design components in the studies were reported to have an appropriate approach to answering the research question, to have utilized appropriate qualitative data collection methods that are adequate to address the research questions, and to have had adequately derived findings from the data. Hence, all studies had a sufficient interpretation of results substantiated by data. All studies proved to have coherence between qualitative data sources, collection, analysis, and interpretation. In one study, the study outcomes of the assessors were not blinded to the intervention, which introduces a risk of bias [20].

In the studies employing quantitative descriptive methodology, three studies reported having a relevant sampling strategy and appropriate measurements in addressing the research question [12,19,36]. While only one study chose a sample that is representative of the population [41], one was not clear [12]. Two studies had a high risk of non-response bias [19,36], while one had a low risk [12]. All three studies utilizing this methodology had an appropriate statistical analysis to answer the research question.

Lastly, all studies reported an adequate rationale for using a mixed methods design to address the research question, having different components of the study effectively integrated to answer the research question, and to have adequately interpreted outputs of the integration of qualitative and quantitative components. Additionally, two of the studies reported to have adequately addressed the divergences and inconsistencies between quantitative and qualitative results [19,20], while two of them did not [12,36]. Lastly, three of the studies had different components of the study adhering to the quality criteria of each tradition of the methods involved [12,19,20] while one study did not [36]. To further assess the findings of this review, we conducted an assessment of the quality of the findings using the GRADE CERQual tool. This is illustrated in Table 7.

#### 3.4.3. Narrative Analysis of GRADE CERQual

Across the synthesis, five core findings were identified, and each was assessed as having moderate confidence using the GRADE-CERQual approach, with confidence ratings largely influenced by methodological and reporting limitations rather than lack of coherence or relevance. The evidence on the foundations of peer navigation, including shared lived experience, trust-building, mentoring, and psychosocial support, was highly consistent across studies and strongly relevant to adolescents living with HIV in sub-Saharan Africa, with rich contributions from multiple contexts; however, confidence was downgraded due to limited reflexivity and unclear integration of qualitative and mixed-methods data in several studies. Evidence relating to training and preparation of PNs was coherent and directly applicable to the review question, but confidence was reduced because many studies provided insufficient detail on training duration, structure, and competency assessment, limiting the adequacy of the data. Findings on supervision and support systems consistently highlighted the importance of emotional and clinical oversight in sustaining peer navigator wellbeing and performance, yet incomplete reporting on supervision frequency, structure, and feedback mechanisms constrained confidence despite agreement across studies. With respect to delivery modalities and flexibility, studies consistently supported the use of in-person, mobile, and hybrid approaches adapted to adolescents’ preferences, although limited reporting on contact intensity, platform use, and fidelity monitoring reduced adequacy and comparability.

Finally, evidence on mechanisms of change underpinning peer navigation, such as trust, emotional support, empowerment, shared experience, and normalizing of ART, was consistently described but largely inferred rather than explicitly measured, resulting in moderate confidence due to limited methodological transparency regarding causal pathways. These mechanisms were generated through inductive thematic synthesis rather than guided by a single explicit theoretical framework. The mechanisms are primarily supported by qualitative participant accounts describing perceived benefits of peer relationships, particularly trust and emotional safety. Other mechanisms, such as empowerment and self-efficacy, were more often inferred from patterns in the data rather than explicitly articulated by participants. As such, they represent analytical interpretations rather than empirically tested causal pathways. Thus, this approach carries limitations, including potential interpretive bias and the absence of formal validation of mechanisms, which should be considered when interpreting the findings. Collectively, these assessments indicate that while the qualitative evidence base for peer navigation and support interventions among adolescents living with HIV in sub-Saharan Africa is coherent and highly relevant, greater methodological clarity, and more detailed reporting are needed to strengthen confidence and support replication, scale-up, and sustainability.

## 4. Discussion

This review highlights how peer navigation and support interventions function among ALHIV in SSA and demonstrates that these interventions achieve both clinical and psychosocial benefits. Across studies, peer navigation and support interventions remained acceptable and consistently improved ART adherence, viral suppression, clinic attendance, and the rates of ART initiation [36,43]. In addition, adolescent self-stigma and isolation were reduced, contributing to improved self-worth, positive body image and strengthened relationships with caregivers [40,41]. Given that stigma and discrimination are associated with reduced ART adherence and treatment uptake among ALHIV [44], peer navigators (PNs), through their role-modelling function, aid in neutralizing ART-related stigma and reduce anxiety in adolescents when navigating complex healthcare pathways [40].

Using the CIMO lens, the synthesis shows that outcomes are not achieved solely through the presence of PNs, but through psychosocial and relational mechanisms such as trust-building. Across studies, PNs built rapport and trust through sharing their lived experiences and relatability, which created a safe space for disclosure, emotional validation, problem-solving and collaboration between PNs and adolescents [40,41]. These mechanisms were associated with increased resilience, reduced stigma and higher self-efficacy as they aided in engagement in care for ALHIV [45]. Where peer-led interventions were supported by structured training, reliable supervision and delivery modes aligned with ALHIV preferences, these mechanisms were more consistently reflected in improved adherence and clinic attendance behaviors [41]. From an implementation perspective, the mechanism’s consistency across varied settings indicates that it represents core functions of peer navigation instead of context-specific features [46]. Although these mechanisms were not always formally tested, the findings suggest that PNs function as a bridge between adolescents’ lived experiences and biomedical care requirements, helping to normalize ART adherence and reduce barriers. This role is particularly important during adolescence, a developmental stage marked by identity formation, school demands and peer influence, whereby stigma and mental health instability persist to undermine adherence across different contexts [20,40].

The findings emphasized the significance of training for safe and high-quality implementation of the peer-led interventions. However, the assessment of training competency, content and educational literacy for PNs varied widely across interventions. Thus, training has occurred over periods of one day to one month across the studies, with content mostly focusing on HIV literacy, confidentiality, mental health, and communication skills. Several studies described training as a once-off activity, while others reported ongoing or refresher training, usually informal and rooted in routine supervision structures [11,21]. Among other things, the content of training PNs across the studies included HIV treatment literacy, communication skills, counselling, active listening, and relationship building. The content also covered ethical principles, particularly confidentiality, boundaries, and appropriate disclosure and psychosocial support (for stigma reduction among LHIV) [9,18,19]. Some studies additionally noted the existence of referral processes and training in health-system navigation, although there were limitations in the depth of the content [17]. Importantly, methods for assessing peer navigator competencies were largely absent or poorly reported across the included studies. With a small number of studies referring to informal assessments, including role-plays during training or observation by the staff in the program, but these lacked standardization and clear detailed reports [28,29]. Most studies also showed a lack of reports in the use of formal competency frameworks for assessments [27]. The limitation of detailed description regarding training sequence, curriculum specification, supervised practice and readiness assessment reduced the level of comparison across interventions and the assessment of training intensity required for complex tasks (e.g., group information management). On the other hand, studies that applied learning approaches such as counselling and role plays consisted of PNs who had greater confidence in problem-solving and non-judgmental communication skills [8,20].

The findings highlight the significance of supervision and structured support for PNs’ well-being and the effectiveness of the interventions. As reported by WHO, incorporating scheduled supervision, problem solving, reflective debriefs, and case reviews aids in maintaining PNs’ quality of service delivery and helps them mitigate burnout [46]. Across studies, supervision appeared as a critical factor in providing problem-solving and emotional support, helping sustain the performance of PNs [16,17,42]. PNs’ supervision appeared to be heterogeneously reported across studies, fluctuating from formal, structured supervision (e.g., meetings scheduled with clinicians) to informal support rooted within routine service delivery [20,40]. Some studies reported mainly clinical supervision, which focused on psychosocial challenges and case discussions, while others focused on administrative supervision, such as coordinating tasks and reporting [27,30]. However, many of the intervention programs utilized supervision only when needed, resulting in limited details on frequency, feedback processes, and modality. Thus, such gaps amplify PNs’ emotional load and increase the chances of inconsistency in practice over time. As such, the inconsistency in reporting of supervision tends to limit conclusions regarding optimal supervision models and the sustainability of long-term programs. Supervision and support as a domain showed moderate confidence across reviews due to inconsistent reporting [47], highlighting a need for supervision that is formalized with explicit intentions, such as support, safeguarding, and quality assurance for a quality program.

Peer navigation and support intervention programs constantly use hybrid delivery, including in-person and digital approaches to the provision of support in alignment with the schooling and mobility of ALHIV [36,39,41]. The success of the interventions is based on supportive supervision for PNs and clarification of roles between HCWs and PNs. In contrast, environments with high stigma, insufficient compensation, inadequate supervision, and emotional burden tend to undermine the continuity, quality, and effects of peer navigation and support intervention programs [20,45]. Such contextual factors aid in explaining why certain interventions tend to produce psychological improvements that do not adequately correspond with improvements in clinical outcomes. Thus, lacking stable structures and satisfactory resources and active mechanisms limiting the sustenance of longer support during the implementation and a lack of effective achievement of viral suppression [20,43].

Regardless of the growing body of literature, the synthesis still identified gaps in the existing evidence base. Firstly, there are still limited trainings that are standardized for PNs, with a lack of training content, competency assessment, duration, and frequency specifications, as many studies were unable to provide sufficient details that permit replication of the trainings. Secondly, the process of implementing and delivering mechanisms is often under-reported, limiting full comprehension of how well the interventions operate in practice [48]. Thirdly, even though psychosocial outcomes are often steadily described, the pathways to sustaining viral suppression still require properly designed and supported peer-led interventions [43]. Lastly, even though PNs play a pivotal role in delivering the interventions, only a few studies explicitly address emotional labor, well-being and sustainability of the workforce provided by PNs [17,46]. Therefore, it is recommended that peer navigation and support interventions should prioritize hybrid (e.g., in-person and online) delivery for ALHIV. It is also recommended that policymakers should make use of these interventions and integrate the roles of PNs into national HIV strategies. Collectively, these actions can further strengthen the quality of the interventions and implementation and sustain the well-being of PNs for the benefit of ALHIV.

### Strengths and Limitations of This Study

This qualitative evidence synthesis employed a systematic and transparent approach, including duplicate screening, the use of established critical appraisal tools, and application of the GRADE-CERQual framework to assess confidence in review findings, which strengthens the methodological rigor of the study. By focusing exclusively on studies conducted in sub-Saharan Africa, the review ensured strong contextual relevance to high HIV-prevalence settings, although this limits the transferability of findings to other regions. The exclusion of non-English publications and grey literature may have resulted in the omission of additional perspectives on peer navigation and support interventions. Furthermore, incomplete reporting of training components, supervision structures, implementation context, and intervention fidelity across included studies limited the ability to fully compare and assess intervention characteristics. Finally, methodological limitations in some primary studies, particularly limited reflexivity, reduced confidence in certain synthesized findings.

## 5. Conclusions

Peer navigation and support interventions are essential components for ALHIV within SSA, as through sharing lived experiences, role modeling, and provision of support by PNs to adolescents, trust is built, ART becomes normalized and resilience is built, thus leading to engagement in care and sustained adherence [40,41]. Despite an evident gap in training and supervision of PNs, cumulative evidence showcases significant psychosocial and clinical benefits of having contextually supported and adapted interventions for ALHIV. Thus, this highlights the significance of having advanced standardized training, fidelity measurements, robust supervision, and policy integration of peer navigation and support intervention programs. Through implementing peer navigation, it can positively contribute to VLS, ART adherence, healthy mental well-being, engagement in care and overall improve the quality of life for ALHIV. In essence, PNs are pivotal to the everyday living of ALHIV, as they serve as role models and support structures. Therefore, we recommend that peer navigation and support interventions be guided by standardized training that clearly describes the frequency of training PNs and core competencies and consists of structured supervision, as this is necessary to ensure the quality of the intervention in various environments and protect the well-being of PNs.

## Figures and Tables

**Table 1:** PCC framework for selection of studies [29].

Research Question: What Are the Experiences and Outcomes of Peer Navigation Interventions to Support Adolescents Living with HIV Treatment in Sub-Saharan Africa?	
P—Population	Adolescents and young adults living with HIV
C—Concept	Peer navigation and support interventions
C—Context	Sub-Saharan Africa

**Table 2:** Characteristics of included studies.

Author (Year)	Aim	Country	Study Design	Study Sample(s)	Data Collection Method	Data Analysis Method	Number of Participants
Hacking (2019) [36]	To determine whether the virtual mentorship program was acceptable to mentors and mentees	South Africa	Mixed methods	Mentees: youth newly diagnosed with HIV. Mentors: stable youths living with HIV and in care.	Routine clinical data: ART initiation, retention, viral load completion/suppression. In-depth interviews with 5 mentors and 5 mentees.	Quantitative: cohort comparisons between mentees and matched controls. Qualitative: thematic analysis	Quantitative: 70 Qualitative: 10
Bakari (2025) [37]	To describe the perceptions of multiple stakeholders on the anticipated role of peer mentors in introducing long-acting antiretroviral therapy (LA-ART) for AYAH into existing comprehensive care programs	Kenya	Qualitative	AYPLHIV, who had been on ART for 11+ years	Focus groups	Thematic analysis	58
Adhiambo (2025) [38]	To document experiences with phone-based peer navigation among AYAH	Kenya	Qualitative	Adolescents 14–19 years. Young adults 20–24 years.	In-depth individual interviews	Thematic analysis	20
Ahonkhai (2023) [19]	To describe the feasibility, acceptability, and adoption of the intervention, and to identify and describe implementation strategies used or adapted to promote intervention success	Nigeria	Mixed methods	Adolescents and young adults living with HIV (AYAH) aged 15–24 years.	Qualitative: informant interviews and focus group discussions. Quantitative: self-reported assessments.	Qualitative: Directed content analysis. Quantitative: Descriptive statistics.	40
Merrill (2023) [31]	To explore youths’ experiences with Project YES!	Zambia	Qualitative sub-study embedded in an RCT	Youth 15–24 years living with HIV.	Semi-structured in-depth interviews and Focus groups	Thematic analysis	40
Ahmed (2022) [16]	To describe adolescent specific service delivery practices used by Expert Clients	Eswatini	Exploratory qualitative descriptive	(1) Expert Clients, (2) ALHIV participating in Teen Club, and (3) key informants (seven nurses, three program managers, and two program coordinators).	Semi-structured interviews and focus group discussions	Thematic analysis	32
Kwena (2024) [39]	To understand the preferences of AYAH on peer navigation strategies	Kenya	Qualitative inquiry	AYAH aged 14–24 years enrolled in HIV care at three clinics in Kisumu County	Focus group discussions	Content analysis	17
Rencken (2021) [40]	To explore the role of peer support in facilitating ART adherence and adoption of strategies to live successfully with HIV among adolescents living with HIV (ALHIV)	South Africa	Qualitative	35 ALHIV aged 12–19 years who receive treatment from a dedicated adolescent clinic, and 35 caregivers of adolescents living with HIV.	Semi-structured interviews	Thematic analysis using an iterative process	70
Atujuna (2021) [41]	To describe the design and delivery of Khuluma, a group-based mHealth intervention, to provide psychosocial support groups to ALWH through the use of peer-led facilitation	South Africa	Pilot intervention study	Adolescents living with HIV (ALHIV) aged 15–20 years.	Focus group discussions	Thematic analysis. Grounded inductive approach to analyze text message data	52
Simms (2022) [20]	To evaluate whether peer counsellors (CATS) trained in problem-solving therapy (PST) could improve virological suppression and mental health outcomes among ALHIV compared with standard peer counselling alone	Zimbabwe	Cluster randomized controlled trial	Adolescents living with HIV aged 10–19 who screened positive for CMD’s.	Clinical assessments: viral load at baseline and 48 weeks. Mental health screening tools: SSQ, PHQ-9 (depression), EQ-5D (health-related quality of life). Qualitative: FGDs and case review notes with peer counsellors and supervisors.	Quantitative: logistic regression with adjustment for clustering at clinic level. Qualitative: thematic analysis.	842
Ahmed (2023) [17]	To elucidate the specific roles and responsibilities of expert clients in service delivery among adolescents living with HIV	Eswatini	Qualitative descriptive	(1) Expert clients; (2) adolescents living with HIV who were current members of the Teen Club; and (3) key informants (program managers, program coordinators and nurses)	Semi-structured interviews, and four focus group discussions and a questionnaire	Content analysis	64
Ferris France (2023) [42]	To explore the perceived impact of Wakakosha Intervention, which is an intervention that aimed to equip AYPLHIV in the use of IBSR/self-inquiry to manage stressful beliefs, reduce self-stigma and grow their sense of self-worth and wellbeing using Thirty Zvandiri Community Adolescent Treatment Supporters.	Zimbabwe	Qualitative	Adolescents and young people living with HIV (AYPLHIV) aged 18–24 years.	Focus group discussion and individual, in-depth interviews	Thematic analysis	62
Zamudio-Haas (2024) [12]	To explore the experiences and perceived impacts of the Zvandiri peer-led treatment support model for young adults recently diagnosed with HIV in Zimbabwe	Zimbabwe	Phenomenology	Young adults living with HIV, recently diagnosed, engaged at the SHAZ! Hub in Chitungwiza.	Semi-structured interview	Interpretive Phenomenological Analysis	45

**Table 3:** Table of characteristics of peer navigation intervention.

Author (Year)	Name of PNs	Description of PNs	Training of PNs	Measures and Procedures of Peer Navigation	Mode of Delivery	Required Outcome of the Intervention
Hacking (2019) [36]	Peer mentors	HIV-positive youths with prior personal struggles with HIV, motivated to help peers.	Virtual mentors underwent a 1-daytraining session	Mentorship was delivered via mobile phones (calls and text). Youth mentors provided encouragement, counselling, and support for clinic attendance. Mentees reported valuing phone calls as a confidential and supportive format. Mentors and mentees discussed barriers such as fear of disclosure, lack of status acceptance, and stigma.	Hybrid	Increased ART initiation (mentees: 80% vs. controls: 42%). Increased viral load completion (80% vs. 45%). No significant difference in viral load suppression or retention in care at 6 or 12 months. Qualitative: mentorship acceptable to mentors and mentees; improved comfort in discussing HIV; reduced isolation.
Bakari (2025) [37]	Peer mentors	Peer mentors, who have lived experiences in a particular situation or condition and can, therefore, provide unique insights and empathy to other persons going through similar experiences.	The administrative structure of the services, fundamental understanding of LA ART and its possible adverse consequences, and its incorporation into current ART programs had been the primary subjects of the peer mentors’ training. Adherence problems related to young people’s interactions with others, such as recreational and academic activities, were a further objective of the training.	Peer mentors were involved in organizing the focus group discussions to ensure that the sessions were lively and youth friendly. Peer mentors were important to the implementation of LA ART for AYLPHIV, (1) for facilitation of communication, (2) for facilitation of referrals, and (3) due to having empathy from their lived experiences.	In-person	Increased knowledge and awareness of integrated services and reduction in concerns about stigma and confidentiality and improved linkage to the services as well as uptake and retention in care.
Adhiambo (2025) [38]	Peer navigator	PNs who were young adults aged 19–28 years living with HIV.	They received a 5-day standardized training on ART adherence counselling, psychosocial support, case management, electronic and in-person navigation, data collection, and entry, or standard of care.	The E-NAV intervention (a combined strategy involving automated SMS and electronic peer navigation support through the use phone calls & social media) consists of an initial in-person meeting followed by bi-weekly sessions for the first two months and subsequently transitioning to monthly sessions. These sessions, which take place over the phone by a peer navigator, aim to address barriers and strengthen resilience among AYAH. The intervention is delivered for one year following study enrolment or until participants are re-randomized to a stage 2 intervention, whichever is sooner. E-NAV participants also receive bi-weekly auto-mated tailored text messages focused on health promotion, visit attendance, and medication adherence. PNs communicate with participants electronically on WhatsApp, Messenger, short message service (SMS), or through a phone call. This is based on the participant’s preference for the method of communication. Each peer navigator, who was not gender specific, had a caseload of approximately 20 participants. Participants are assigned to navigators who had the least caseloads at the time. The facility-based PNs are supervised by a Navigator Coach, with counselling experience, and trained in providing psychosocial support and HIV education targeting ART and clinic attendance adherence.	Hybrid	Participants are followed up for two years for the primary outcome of viral suppression and retention in HIV care and treatment. Reported E-NAV benefits included adherence and appointment reminders, increased knowledge about HIV care, and strategies to address HIV stigma.
Ahonkhai (2023) [19]	Peer navigators	Non-patient key stakeholders (5 male, 4 female). Highly motivated individuals who were already invested in providing peer support and had valuable experience in behaviors and skills associated with successful ART adherence.	PNs received an intensive two-day training prior to study commencement. The curriculum for this training was developed with input from youth and healthcare providers in the HIV clinic and included didactic sessions, group activities, telephone call role-play, and hands-on demonstration of the short message service (SMS) reminders. Topics included an introduction to HIV/AIDS, a discussion of common myths, ethical issues related to HIV care, privacy and confidentiality, professionalism, HIV prevention strategies, basic counselling and communication skills, telephone etiquette, mental health among AYAH, sexual health and youth-friendly services.	PNs were recruited from engaged peer leaders in the HIV clinic where the intervention was based. This helped the study team identify highly motivated individuals who were already invested in providing peer support and had valuable experience in behaviors and skills associated with successful ART adherence.	Hybrid	We used explanatory, mixed methods to assess implementation outcomes (feasibility, acceptability, and adoption) and identify implementation strategies used or adapted to promote intervention success (ART adherence).
Merrill (2023) [31]	Youth Peer Mentor (YPM)	YPM, aged 21–26 years, who were identified by healthcare providers (HCP) in the four study clinics as successfully managing their HIV.	Completed a two-week training and were hired by Project YES! to work in the clinics. They underwent one month of practice meetings with youth before the intervention’s launch.	Participants randomized to the intervention arm received a six-month peer-mentoring program, which included an orientation meeting (with optional caregiver participation) and monthly individual and monthly group meetings with a youth peer mentor (YPM).	In-person	Increased motivation for care, improved ART adherence and, for some, virologic results; reduced shame/self-stigma; strengthened connection and community.
Ahmed (2022) [16]	Expert clients (ECs)	Expert clients are HIV-positive lay health workers known to provide psychosocial support to their seropositive peers.	Expert clients receive a week-long training comprising of the role of ECs, communication and counselling skills, and other HIV-related content such as HIV treatment adherence, stigma and disclosure, and HIV linkage to care.	Expert clients are the bridge between the community and the healthcare system, and, therefore, are critical for reengaging adolescents who are lost to follow-up in HIV care. Additionally, expert clients have the potential to prevent HIV reinfection and onward transmission among adolescents through the provision of community-based adherence support and sexual health education.	In-person	To promote resilience and independence in HIV treatment among adolescents.
Kwena (2024) [39]	Peer navigators	Characteristics desired of a navigator are a person of the same age group and HIV status who has a good memory, patience, is encouraging, friendly, and able to maintain confidentiality.	Not stated.	AYAH preferred interventions delivered through secure communication platforms (e.g., WhatsApp, phone calls, and text messaging), with status-neutral messages and emphasis on confidentiality and relationship-building.	Hybrid	Formative preferences to inform A4A design aimed at retention and improved treatment outcomes among AYAH.
Rencken (2021) [40]	Lay Counsellor	Lay counsellors trained in adolescent care.	Trained in adolescent care, and adolescent attendance in the group is high.	All ALHIV receiving treatment from the HIV clinic were invited to participate in a peer-support group, which ran weekly on days designated for adolescent clinic visits. The peer-support group is facilitated by a lay counsellor trained in adolescent care, and adolescent attendance in the group is high. Discussion topics among peers include living well with HIV, disclosure to partners or friends, and safer sexual practices.	In-person	Peer support encouraged adherence to ART. The peer support group fostered peer relationships, and caregivers perceived increased self-acceptance and adherence.
Atujuna (2021) [41]	Peer mentors	The peer mentors were ALWH from both provinces trained via a counsellor-led workshop on group facilitation and use of the technology platform.	The training workshop, which all facilitators attended prior to study implementation, was designed by the SHM Foundation, adapted from the Positive Connections manual developed by FHI360 for leading information and support groups for adolescents living with HIV. Facilitators were briefed on key issues facing this population, including HIV and AIDS knowledge, adherence to ART, stigma, disclosure, socioeconomic issues, gender-based violence and education. They were trained on facilitation skills and techniques such as active listening, empathetic encouragement, non-judgmental communication, asking open ended questions, validation and problem solving. They were encouraged to draw on their own experiences as parents, friends, siblings or caregivers in order to develop their skills. The facilitators were briefed on the model and technological platform. This involved imparting skills in trouble shooting and referral processes. The final part of the workshop involved scenario-based thinking, where participants were given difficult issues to engage and resolve that might occur in the groups, such as conflict between participants, non-participation, suicidal ideation or a participant spreading a myth. Facilitators were given a training manual that included the subjects covered in this workshop.	These support groups were virtually enabled through a digital platform, where participants discuss—peer-to-peer, at anytime, anywhere via text message—a range of issues pertinent to their needs. Active facilitation of group sessions utilizing the Khuluma curriculum was held Monday to Friday from 3 to 7 p.m. Critically, the model was peer-led, with trained peer mentors facilitating conversations, supported at all times by trained professional counsellors. Participants chose a pseudonym, allowing them to remain anonymous and communicate in small groups.	Online	Positive health and mental health outcomes for populations experiencing stigma and social isolation.
Simms (2022) [20]	Community Adolescent Treatment Supporters (CATS).	CATS were young people aged 18–24 years, living with HIV, trained to deliver peer support.	CATS received structured PST training, adapted from the Friendship Bench model: Core PST steps (problem identification, brainstorming solutions, action planning). Counseling skills and empathic listening. Role-plays and practice sessions. Ongoing supervision by trained mental health professionals and senior counsellors.	Control group: standard Zvandiri peer counselling (adherence, psychosocial support, linkage to care). Intervention group: CATS additionally delivered PST sessions, focusing on discussing personal problems and working through structured problem-solving steps. Regular case reviews with supervisors. CATS documented each session and escalated red-flag cases (e.g., suicidal ideation).	Hybrid	Primary outcome: virological non-suppression at 48 weeks (VL ≥ 1000 copies/mL). Secondary outcomes: changes in CMD symptoms (SSQ score), depressive symptoms (PHQ-9), and health-related quality of life (EQ-5D). Qualitative outcomes: feasibility, acceptability, and fidelity of CATS delivering PST.
Ahmed (2023) [17]	Expert clients	Expert clients often work alongside nurses to facilitate Teen Clubs, peer support groups widely available for ALHIV in countries throughout sub-Saharan Africa.	Expert clients had varying degrees of skills, training and/or resources	Adherence support consisted of counselling, education, follow-up visits and pill counts (i.e., monitoring adherence by calculating the number of pills taken since the last refill appointment). Community-based ECs were primarily responsible for tracking clients who had missed a clinic appointment within seven to ninety days (i.e., defaulters) or over ninety days (i.e., lost to follow-up) by visiting their homes and, if found, encouraging them to resume treatment and attend their appointments. On the other hand, facility-based Expert Clients performed all of their work at a health facility, which includes conducting counselling sessions and pill counts, following up with patients via phone calls and documenting patient information. Facility-based clients would refer defaulters to community-based Expert Clients for home-based follow-up. Community-based Expert Clients would also consult with other community health workers (e.g., rural health motivators) to locate defaulters or clients that were lost to follow-up.	In-person	Improve retention in care and treatment adherence.
Ferris France (2023) [42]	Community Adolescent Treatment Supporters (CATS).	Community Adolescent Treatment Supporters are 18–24 years-olds LHIV, trained, mentored and supported to deliver structured support groups, counselling and tailored community-based adherence support to their peers.	Coaches trained in IBSR methods, mindfulness, meditation, creativity, facilitation, journaling.	16 × 3-h group sessions. Supported by a 156-page activity journal. Sessions included self-inquiry, mindfulness, meditation, body work, creativity.	Hybrid	Reduce HIV self-stigma, improve self-worth, agency, communication, relationships, body positivity, and emotional wellbeing.
Zamudio-Haas (2024) [12]	Community Adolescent Treatment Supporters (CATS).	Young people living with HIV, aged 18–24 (themselves HIV-positive), trained as peer counsellors.	Community Adolescent Treatment Supporters trained by Africaid/Zvandiri to provide: HIV education and treatment literacy, Psychosocial support, Case management and accompaniment to clinics, Guidance on disclosure.	Community Adolescent Treatment Supporters played a pivotal role for youth, providing emotional, educational, and logistical support to facilitate treatment initiation, adherence, and persistence in care. The Community Adolescent Treatment Supporters program supported youth through multiple approaches: group sessions, individual meetings, and via text or phone. While Community Adolescent Treatment Supporters offered counselling and comfort to participants, they emphasized the long-term importance of identifying at least one other person in participants’ lives who could know their status and support them around HIV.	Hybrid	Community Adolescent Treatment Supporters facilitated linkage and retention in care. Community Adolescent Treatment Supporters work between the homes, health facilities, and mobile platforms to support case management and health education for children and young people in need of HIV services.

**Table 4:** Theme linked to the CIMO Framework.

Major Theme Category	Sub-Themes/Codes to Explore	Analytical Focus (for Synthesis)	Links to CIMO (Mechanisms/Context)
1. Foundations of Peer Navigation	Roles & Responsibilities of PNs Why PNs matter (peer relatability, role models) Qualities of effective PNs	Define what peer navigation is across contexts	Intervention (I)—Composition and identity of PN workforce
2. Training & Preparation	Training content & length Lack of standardized manuals Competency development (HIV literacy, counselling, confidentiality)	How PNs are equipped to deliver interventions	Mechanism Enabler—Skill-building before delivery
3. Supervision & Support Systems	Case reviews/clinical oversight Emotional burden & burnout Supportive supervision vs. abandonment	Sustainability and PN wellbeing	Context (C)—Implementation conditions
4. Delivery Modalities & Flexibility	Mode: In-person, mobile, hybrid Frequency: weekly/monthly calls/messages Participant preferences (phone vs. clinic)	Adaptation to youth needs & lifestyles	Intervention Design (I)—Mode affects activation of mechanisms
5. Mechanisms of Change	Trust and rapport-building Shared lived experience Social and emotional support Disclosure coaching & adherence problem-solving	Why peer navigation works beyond routine care	Mechanism (M)—Psychological/social processes
6. Outcomes & Impact	ART adherence & clinic attendance Viral load suppression Self-worth & stigma reduction Resilience/independence	Evidence of PN effectiveness (clinical & psychosocial)	Outcome (O)—What interventions achieve

**Table 5:** CASP Results.

Study ID	1. Was There a Clear Statement of Aims and Objectives of the Research?	2. Is a Qualitative Methodology Appropriate?	3. Was the Research Design Appropriate to Address the Aims of the Research?	4. Was the Recruitment Strategy Appropriate to the Aims of the Research?	5. Was the Data Collected in a Way that Addressed the Research Issue?	6. Has The Relationship Between the Researcher and Participants been Adequately Considered?	7. Have Ethical Issues Been Taken into Consideration?	8. Was the Data Analysis Sufficiently Rigorous?	9. Is There a Clear Statement of Findings?	10. How Valuable is the Research?
Adhiambo et al. (2025) [38]	Yes	Yes	Yes	Yes	Yes	Yes	Yes	Yes	Yes	Yes
Merrill (2023) [31]	Yes	Yes	Yes	Yes	Yes	Can’t Tell	Yes	Yes	Yes	Yes
Kwena et al. (2024) [39]	Yes	Yes	Yes	Yes	Yes	Yes	Yes	Yes	Yes	Yes
Rencken et al. (2021) [40]	Yes	Yes	Yes	Yes	Yes	Can’t Tell	Yes	Yes	Yes	Yes
Ahmed et al. (2022) [16]	Yes	Yes	Yes	Yes	Yes	Can’t Tell	Yes	Yes	Yes	Yes
Ahmed et al. (2023) [17]	Yes	Yes	Yes	Yes	Yes	Can’t Tell	Yes	Yes	Yes	Yes
Ferris France et al. (2023) [42]	Yes	Yes	Yes	Yes	Yes	Can’t Tell	Yes	Yes	Yes	Yes
Bakari et al. (2025) [37]	Yes	Yes	Yes	Yes	Yes	Can’t Tell	Yes	Yes	Yes	Yes
Zamudio-Haas et al. (2024) [12]	Yes	Yes	Yes	Yes	Yes	Can’t Tell	Yes	Yes	Yes	Yes
Can’t tell: refers to insufficient detail reported by the authors to permit judgment. APPRAISAL SUMMARY: List key points from your critical appraisal that need to be considered when assessing the validity of the results and their usefulness in decision-making.										
Study ID		Positive/Methodologically sound			Negative/Relatively poor methodology			Unknowns		
Adhiambo (2025) [38]								Recruitment strategy not explicitly stated		
Merrill (2023) [31]					Reflexivity is limited					
Kwena (2024) [39]					Small sample size					
Rencken (2021) [40]					Reflexivity is limited					
Zamudio-Haas (2024) [12]					Reflexivity is limited					

**Table 6:** MMAT table.

Category of Study Designs	Methodological Quality Criteria	Atujuna et al. (2021) [41]	Ahonkhai et al. (2023) [19]	Hacking et al. (2019) [36]	Simms et al. (2022) [20]
Screening questions (for all types)	S1. Are there clear research questions?	Yes	Yes	Yes	Yes
	S2. Do the collected data allow to address the research questions?	Yes	Yes	Yes	Yes
1. Qualitative	1.1. Is the qualitative approach appropriate to answer the research question?	Yes	Yes	Yes	Yes
	1.2. Are the qualitative data collection methods adequate to address the research question?	Yes	Yes	Yes	Yes
	1.3. Are the findings adequately derived from the data?	Yes	Yes	Yes	Yes
	1.4. Is the interpretation of results sufficiently substantiated by data?	Yes	Yes	Yes	Yes
	1.5. Is there coherence between qualitative data sources, collection, analysis, and interpretation?	Yes	Yes	Yes	Yes
2. Quantitative randomized controlled trials	2.1. Is randomization appropriately performed?				Yes
	2.2. Are the groups comparable at baseline?				Yes
	2.3. Are there complete outcome data?				Yes
	2.4. Are outcome assessors blinded to the intervention provided?				No
	2.5 Did the participants adhere to the assigned intervention?				Yes
3. Quantitative non-randomized	3.1. Are the participants representative of the target population?				
	3.2. Are measurements appropriate regarding both the outcome and intervention (or exposure)?				
	3.3. Are there complete outcome data?				
	3.4. Are the confounders accounted for in the design and analysis?				
	3.5. During the study period, is the intervention administered (or exposure occurred) as intended?				
4. Quantitative descriptive	4.1. Is the sampling strategy relevant to address the research question?	Yes	Yes	Yes	
	4.2. Is the sample representative of the target population?	Yes	No	Can’t Tell	
	4.3. Are the measurements appropriate?	Yes	Yes	Yes	
	4.4. Is the risk of nonresponse bias low?	Yes	No	No	
	4.5. Is the statistical analysis appropriate to answer the research question?	Yes	Yes	Yes	
5. Mixed methods	5.1. Is there an adequate rationale for using a mixed methods design to address the research question?	Yes	Yes	Yes	Yes
	5.2. Are the different components of the study effectively integrated to answer the research question?	Yes	Yes	Yes	Yes
	5.3. Are the outputs of the integration of qualitative and quantitative components adequately interpreted?	Yes	Yes	Yes	Yes
	5.4. Are divergences and inconsistencies between quantitative and qualitative results adequately addressed?	Can’t Tell	Yes	Can’t Tell	Yes
	5.5. Do the different components of the study adhere to the quality criteria of each tradition of the methods involved?	Yes	Yes	Can’t Tell	Yes

Can’t tell: refers to insufficient detail reported by the authors to permit judgment.

**Table 7:** GRADE CERQual Assessment.

Summary of Review Finding	Studies Contributing to Review Findings	Methodological Limitations	Coherence	Adequacy	Relevance	CERQual Assessment	Explanation of Assessment
Foundations of peer navigation	[12,16,17,19,20,36,37,38,39,41,42].	Moderate methodological limitations: The consideration of the relationship between researcher and participant is unclear in 7 studies. One study [36] did not clearly combine the qualitative and quantitative results of the data. Two studies [16,41] had unclear methodological consistencies.	No or very minor concerns: The data has no variability in terms of the foundations and understanding of peer navigation.	No or very minor concerns: All included studies contributed to this finding.	No or very minor concerns: All studies are relevant to the research question of this review and accurate to this finding.	Moderate confidence	This finding was graded as moderate as a result of concerns regarding methodological limitations in 7 of the included studies.
Training and Preparation	[12,16,17,19,20,31,36,37,38,39,40,41,42].	Minor to moderate limitations. Some studies reported unclear training and preparation of peer navigators [16,17,18,21,37,39,41,42]. This led to lack of detailed training content and delivery among other studies, such as [16,17,37,39,41,42], which limited the quality assessment of the implementation process.	No or very minor concerns: The findings were consistent across all studies.	Moderate coherence: All included studies contributed to this finding. However, the lack of detailed training intensity and structure limits the adequacy and richness of the training methods.	High relevance: All studies focused directly on the preparation and training of PNs for ALHIV.	Moderate confidence	The studies reported moderate confidence because the variation in duration of training and reporting was unclear in some of the studies.
Supervision and support systems	[12,16,17,20,37,39,41,42].	Minor to moderate limitations. Several studies did not provide clear frequency of supervision and description of the supervision [16,17,22,37,42]. The support system and feedback processes were not properly stated in several studies [16,17,37,39,42].	Moderate coherence: Most of the studies agreed on the essentiality of supervision for emotional well-being and performance of PNs on the interventions. However, some studies reported support to be provided when needed, without a clear structure or fixed schedule of support provision, with some varying widely (e.g., some of the studies worked on a weekly check-in, while others had no frequency of support provision reported).	Moderate adequacy: The data showed adequacy in several studies however there was a lack of detail in the supervision techniques used.	Moderate adequacy: Almost all of the studies contributed directly to the PNs supervision even though some studies did not explicitly address how PNs’ supervision was carried out in supporting ALHIV.	Moderate confidence	The finding was graded as moderate as a result of incomplete reporting on the structure of supervision and support system, but it should be noted that consistent emphasis on the significance of supervision strengthens the findings credibility.
Delivery modalities and flexibility	[12,16,17,19,20,31,36,37,38,39,40,41,42].	Minor to moderate limitations. Several studies did not provide a comprehensive detail on delivery modality (e.g., WhatsApp/SMS) and intensity and frequency of contact between ALHIV and PNs [16,17,37,39,42]. There was also a lack of tracked fidelity, as the sometimes-reported flexibility (e.g., youth-friendly hours) was stated but not tracked or operationalized.	Moderate coherence: All studies support utilizing mixed delivery channels (e.g., SMS) and a flexible engagement schedule. However, missing detailed information on the variability of these modalities and their flexibility across the studies limits the comparability of studies.	Moderate adequacy: More studies contributed to the modes of delivery and flexibility evidence. However, several studies showed limited detail in the specificity of duration and frequency of the implementation, delivery and its monitoring, which limits the depth of the findings.	No or very minor concerns: All studies are relevant to the research question of this review and accurate to this finding.	Moderate confidence	The findings showed multiple deliveries and flexible support engagement to ALHIV. However, there were limited reporting of the frequency of provided support and use of platforms in some studies.
Mechanisms of change	[12,16,17,19,20,37,41].	Minor to moderate limitations: Some of the studies did not provide details on the utilised tools to identify or measure the mechanisms, e.g., [17,18,37]. Some of the studies showed that mechanisms were inferred as compared to being explicitly tested [21,41].	Moderate coherence: The studies showed implicit description, which made it difficult to compare across studies; the studies constantly suggested mechanisms such as trust-building, emotional support, shared lived experience, and empowerment.	Moderate adequacy: Multiple studies showed evidence of data adequacy. However, the depth varied across the studies, with few studies providing rich qualitative data that elaborated as to why these mechanisms led to the engagement and adherence improvement.	High relevance: All of the studies were linked to PN and ALHIV.	Moderate confidence	The findings were graded as moderate due to constant mentioning of the mechanisms, even though the mechanisms were not well measured or operationalized, which limits the study’s certainty of causal pathways.
Confidence level		Description					
High		Refers to strong confidence that reflects the phenomenon of interest.					
Moderate		Refers to little concern about one or more CERQual components that reduce certainty.					
Low		Limited confidence due to the existence of concerns in methodological limitations, coherence, adequacy, or relevance limits confidence in the finding.					
Very low		Very little confidence that the finding reflects the phenomenon of interest.					

## Data Availability

No new data were created or analyzed in this study.

## Abbreviations

VLS	Viral load suppression
SSA	Sub-Saharan Africa
ALHIV	Adolescents living with HIV
PN	Peer navigation
PNs	Peer navigators
